# The effect of oppositional parietal transcranial direct current stimulation on lateralized brain functions

**DOI:** 10.1111/ejn.13086

**Published:** 2015-11-11

**Authors:** Lucia M. Li, Rob Leech, Gregory Scott, Paresh Malhotra, Barry Seemungal, David J. Sharp

**Affiliations:** ^1^ Computational, Cognitive and Clinical Neuroimaging Laboratory (C3NL)Division of Brain SciencesDepartment of MedicineImperial College LondonC3NL 3rd Floor Burlington Danes BuildingDu Cane RoadFulhamLondonW12 0NNUK; ^2^ Centre for Restorative NeuroscienceImperial College LondonLondonUK; ^3^ Neuro‐Otology UnitDivision of Brain SciencesImperial College LondonLondonUK

**Keywords:** non‐invasive brain stimulation, numerical processing, parietal cortex, spatial attention, sustained attention

## Abstract

Cognitive functions such as numerical processing and spatial attention show varying degrees of lateralization. Transcranial direct current stimulation (tDCS) can be used to investigate how modulating cortical excitability affects performance of these tasks. This study investigated the effect of bi‐parietal tDCS on numerical processing, spatial and sustained attention. It was hypothesized that tDCS would have distinct effects on these tasks because of varying lateralization (numerical processing left, spatial attention right) and that these effects are partly mediated by modulation of sustained attention. A single‐blinded, crossover, sham‐controlled study was performed. Eighteen healthy right‐handed participants performed cognitive tasks during three sessions of oppositional parietal tDCS stimulation: sham; right anodal with left cathodal (RA/LC); and right cathodal with left anodal (RC/LA). Participants performed a number comparison task, a modified Posner task, a choice reaction task (CRT) and the rapid visual processing task (RVP). RA/LC tDCS impaired number comparison performance compared with sham, with slower responses to numerically close numbers pairs. RA/LC and RC/LA tDCS had distinct effects on CRT performance, specifically affecting vigilance level during the final block of the task. No effect of stimulation on the Posner task or RVP was found. It was demonstrated that oppositional parietal tDCS affected both numerical performance and vigilance level in a polarity‐dependent manner. The effect of tDCS on numerical processing may partly be due to attentional effects. The behavioural effects of tDCS were specifically observed under high task demands, demonstrating the consequences of an interaction between stimulation type and cognitive load.

## Introduction

Cognitive functions demonstrate varying degrees of hemispheric lateralization (Penfield & Jasper, 1954; Wada & Rasmussen, 1960; Milner, 1971; Desmond *et al*., 1995; Gazzaniga, 2000; Wang *et al*., 2014). Lateralized pathology within the parietal lobes produces distinct cognitive problems depending on the side affected. Impairments of numerical processing and dyscalculia are often produced by parietal lesions in the dominant hemisphere (Göbel *et al*., 2001; Gruber *et al*., 2001; Sandrini *et al*., 2012; Rivera *et al*., 2005; Cantlon *et al*., 2006; Grabner *et al*., 2007; Price & Ansari, 2011; Zukic *et al*., 2012). In contrast, impairments of spatial attention and spatial neglect are most commonly observed following right parietal lesions (Mort *et al*., 2003; Malhotra *et al*., 2009; Corbetta & Shulman, 2011). These impairments have primarily been explained in terms of disruption of a specific cognitive process (Sandrini *et al*., 2012; Bird *et al*., 2006; Corbetta & Shulman, 2011; Zukic *et al*., 2012). However, attention also non‐specifically affects performance on a broad range of tasks, as an adequate ‘intensity’ of attention is often required for efficient goal‐directed behaviour (Parasuraman, 1998).

Some cognitive problems may result from a combination of a specific impairment and a change in attentional processing. For example, spatial neglect after right parietal stroke appears to result from a combination of specific impairments in spatial processing in addition to impaired sustained attention (Husain *et al*., 1997; Husain & Rorden, 2003; Malhotra *et al*., 2009; Corbetta & Shulman, 2011; Langner & Eickhoff, 2013). In addition, attentional measures correlate with arithmetic performance in healthy children, and attentional deficits have been demonstrated in patients with developmental dyscalculia, previously considered a syndrome of pure dyscalculia (Askenazi & Henik, 2010; Anobile *et al*., 2013; Barnes & Raghubar, 2014). Sustained attention can be measured by assessing vigilance level, that is the ‘intensity’ of attention at a particular time (Robertson *et al*., 1997; Sarter *et al*., 2001; Alexander *et al*., 2005; Bonnelle *et al*., 2011). This can fluctuate from moment to moment, for example, if one's mind wanders away from the task. Sustained attention can also be measured by testing for a vigilance decrement, which is a progressive drop in the ‘intensity’ of attention (Malhotra *et al*., 2009; Steinborn *et al*., 2009; Bonnelle *et al*., 2011). This often occurs if one becomes tired or bored with a monotonous task. Previously vigilance has been studied using the choice reaction time task (CRT), a simple speeded response task. Participants often perform the task with low error rates and reaction times (RTs) initially, but may show reduced vigilance level and therefore a vigilance decrement by the end of the task, particularly following brain injury (Bonnelle *et al*., 2011).

Transcranial direct current stimulation (tDCS) has been increasingly used as a non‐invasive and safe technique for studying and modulating many cognitive functions (Fregni *et al*., 2005a,b; Hummel *et al*., 2005; Brunoni *et al*., 2011; Stagg *et al*., 2013), including attention (Boggio *et al*., 2007a,b; Coffman *et al*., 2012; Kang *et al*., 2012; Tseng *et al*., 2012; Weiss & Lavidor, 2012; Nelson *et al*., 2013), working memory (Fregni *et al*., 2005a,b; Marshall *et al*., 2005; Boggio *et al*., 2006; Berryhill *et al*., 2010; Jacobson *et al*., 2012b) and executive function (Fecteau *et al*., 2007; Dockery *et al*., 2009; Boggio *et al*., 2007b; Weiss & Lavidor, 2012). Scalp electrodes are used to apply weak electrical currents to the brain and transiently alter cortical excitability. Anodal stimulation is thought to increase cortical excitability under the electrode, while cathodal stimulation decreases cortical excitability (Nitsche & Paulus, 2000; Jang *et al*., 2009; Stagg & Johansen‐berg, 2012).

An oppositional brain stimulation montage delivers anodal stimulation over one region and cathodal stimulation over the homologous region of the other hemisphere. The expected effect of this montage is to shift the balance of hemispheric activity. Anodal stimulation facilitates one hemisphere whilst cathodal stimulation suppresses the other (Jacobson *et al*., 2012a). This can alter the interaction between hemispheres. Following motor stroke, the contralesional motor cortex is thought to impair recovery by inhibiting the ipsilesional motor cortex. Studies manipulating hemispheric interactions by targeting cathodal stimulation to the contralesional hemisphere have shown functional improvements (Ward *et al*., 2003; Fregni *et al*., 2005a,b; Boggio *et al*., 2007a,b; Sparing *et al*., 2009; Stagg *et al*., 2013). There are limitations to using oppositional montages, as it is difficult to distinguish the effects of facilitation and inhibition. However, the approach has proved useful where the aim is to change the balance of hemispheric activity (Bardi *et al*., 2013), and oppositional stimulation provides its own internal control, as behavioural effects that interact with the polarity of stimulation are potentially easier to separate from non‐specific effects of stimulation (Cohen Kadosh *et al*., 2010; Hecht *et al*., 2010).

Here, the use of oppositional stimulation was extended by performing a single‐blinded, crossover, sham‐controlled study of oppositional parietal tDCS on two cognitive tasks with distinct cortical lateralization: a left lateralized number comparison task (Göbel *et al*., 2001; Gruber *et al*., 2001; Sandrini *et al*., 2012; Rivera *et al*., 2005; Cantlon *et al*., 2006; Grabner *et al*., 2007; Price & Ansari, 2011); and right lateralized modified Posner task used to assess spatial attention (Corbetta & Shulman, 2002; Bird *et al*., 2006; Malhotra *et al*., 2009). It was predicted that the effect of tDCS on the task would reflect its lateralization, that is that right anodal/left cathodal (RA/LC) stimulation should shift the balance of hemispheric activity to the right and improve spatial processing (Posner task) and disrupt numerical processing (number comparison task), whilst right cathodal/left anodal (RC/LA) stimulation should shift the balance to the left hemisphere and have the opposite behavioural effects. Also, the effect of stimulation on vigilance level and decrement was investigated using the CRT and the rapid visual processing task (RVP from the Cantab^®^ battery; Posner *et al*., 1978, 1980; Jones *et al*., 1992; Fan *et al*., 2002; Gau & Huang, 2014). This allowed to test whether any observed effects of stimulation on spatial or numerical processing might be mediated through a non‐specific effect on sustained attention.

## Materials and methods

### Participants

Eighteen healthy controls (nine male, nine female) were recruited (age 20–42 years, interquartile range 21–28 years). All but one of the participants was naïve to tDCS. All participants were right‐handed according to the Edinburgh Handedness Inventory scale (Oldfield, 1971), educated to degree level or above, with no history of neurological or psychiatric illness. Participants gave written informed consent. The study conforms to the Declaration of Helsinki (World Medical Association, 2004). Ethical approval for the study was granted through the local ethics board (NRES Committee London – West London & GTAC).

### tDCS and testing protocol

**Figure 1:**
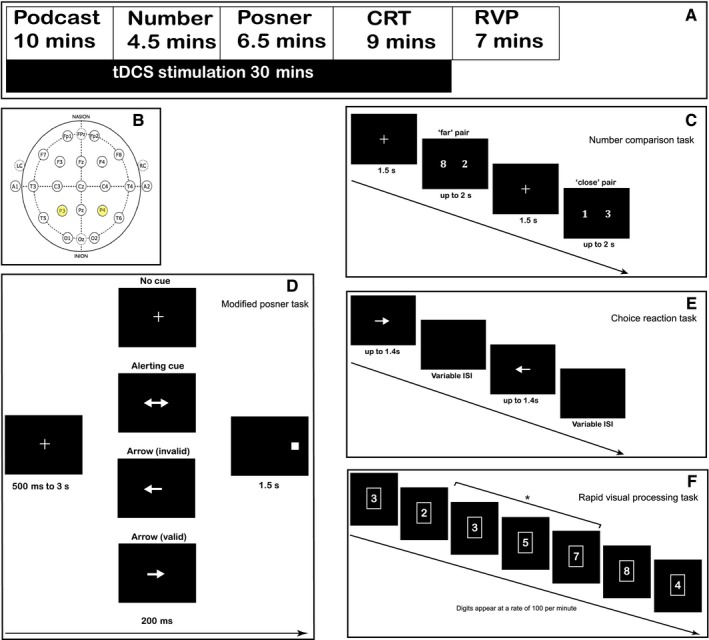
(A) Schema of each testing session. (B) Electrodes were placed at positions P3 and P4 (highlighted) of the international EEG system. (C) Number comparison task: participants saw pairs of single digits and pressed a right or left button to indicate the side of the numerically bigger number. Number pairs were either numerically far, for example 8 and 2, or close, for example 1 and 3. (D) Modified Posner task: participants saw a fixation cross followed by one of three possible cues or the absence of a cue (‘No Cue’). A double‐headed arrow cue alerted the participant to the imminent appearance of a target (‘Alerting Cue’). In two other conditions participants saw arrows that correctly or incorrectly signalled the direction of the target (‘Valid Cue’ and ‘Invalid Cue’, respectively). Participants made a left or right finger press to indicate the position of the target. (E) CRT: participants responded with a left or right finger press to left or right pointing arrows appearing at variable ISIs. (F) RVP from the Cantab^®^ battery: participants saw a continuous stream of numbers and pressed a button when one of three possible pre‐specified three‐digit sequences appeared (e.g. 3–5–7), denoted by *. The response was only valid if the button was pressed in response to the final digit of the sequence (‘7’ in this example).

Each participant attended four testing sessions (Fig. 1A). In the first session, participants practised each task to minimize learning effects during subsequent sessions. During the next three sessions participants received 30 min of tDCS or sham stimulation, separated by a minimum of a 48‐h gap. Each participant had sessions at a similar time of day (i.e. morning or afternoon). The order of sessions was pseudorandomized and counterbalanced across participants. A minimum of 3 min of tDCS is required to produce excitability changes in the motor cortex (Nitsche & Paulus, 2000). No cognitive studies have specifically addressed the minimum duration of stimulation required, but most stimulate for at least 5–10 min prior to the onset of tasks. Therefore, the tasks started after 10 min of stimulation, during which participants listed to an audio podcast. The podcast was different at each session, but the same three podcasts were used in the same order for all participants.

tDCS was delivered using the Magstim HDCKit (Magstim, UK). Silicon electrodes (5 × 5 cm) in water‐soaked cellulose sponges were applied to the scalp with electrode gel, at P3 (left hemisphere) and P4 (right hemisphere) of the 10–20 international EEG system (Fig. 1B; Kim *et al*., 2007). Three montages were used: anode on P4 with cathode on P3 (RA/LC); cathode on P3 with cathode on P4 (RC/LA); or sham stimulation with the RA/LC montage. During real stimulation, the current was ramped on over 30 s, to 2 mA. During sham stimulation, current initially flowed as normal but switched off after 30 s.

### Tasks

Participants performed four cognitive tasks during each session in the same order: (1) a number comparison task; (2) a modified Posner task; (3) a CRT; and (4) the RVP. The RVP was performed on the Cantab^®^ system (button‐press response pad). All other tasks were programmed in MatLab^®^ using Psychtoolbox, and performed on a Macintosh MacBook laptop (13 inch screen), with a separate button‐press response pad with left and right response buttons. Participants were instructed to respond as quickly and as accurately as possible, and response accuracy and RTs were recorded. All statistical analyses were carried out in SPSS (v21; IBM, Armonk, NY, USA). Repeated‐measures anova was used to investigate the effect of tDCS condition on task performance, and to investigate interactions between tDCS condition and task features.

### Number comparison task

A number comparison task assessed numerical processing (Sandrini *et al*., 2012; Fig. 1C). Five practice trials were followed by three blocks of 36 trials. Each trial started with a central fixation cross lasting 1.5 s, followed by presentation of a pair of single‐digit numbers. Each number subtended a visual angle of 0.6° width and 1.7° height, at a visual angle of 1.7° from centre. Participants were required to decide which was the numerically bigger number (left or right). Participants had a maximum of 2 s in which to respond, after which another trial started. All possible single‐digit numbers except ‘5’ and ‘0’ were used, in all possible pairings. Each pairing was presented randomly and twice during each block, with the side of the numerically bigger number counterbalanced. Pairs were deemed ‘close’ if their numerical difference was ≤ 3 and ‘far’ if their numerical difference was ≥ 4. A previous study found that RTs to ‘close’ pairs were longer than to ‘far’ pairs (Sandrini *et al*., 2012). Therefore, accuracy and RTs for ‘close’ and ‘far’ pairs were analysed separately, and the interaction between pair type and tDCS stimulation was tested.

### Modified Posner task

The modified Posner cueing task probed spatial attention (Fig. 1D). Five practice trials were followed by five blocks of 32 trials (Posner *et al*., 1978). Each trial consisted of a central fixation cross, presented for an interval of 500 ms to 3 s, after which one of four conditions was possible. In the first condition, a square target appeared on the right or left of the screen without any other stimulus (‘No Cue’ condition). Participants responded left or right depending on the spatial location of the target. In the other three conditions, an arrow pointing left, right or in both directions was presented centrally for 200 ms prior to the target. A double‐headed arrow encoded no information about the location of the subsequent target, but alerted the participant to imminent target appearance (‘Alerting Cue’ condition; Fan *et al*., 2002). In contrast, the directional arrows accurately cued the spatial location of the subsequent cue 80% of the time (‘Valid Cue’ condition). In 20% of trials, the target would appear on the opposite side (‘Invalid Cue’ condition). The arrows subtended a visual angle of 2.9°. The square targets subtended a visual angle of 1.15° and were located at a visual angle of 5.7° to the right or left of centre. Participants had 1.5 s in which to respond, after which a new trial began. Accuracy and RT were analysed for the four cue conditions, and interactions between tDCS and cue condition were tested.

### CRT

The CRT is a speeded response task measuring information‐processing speed and sustained attention (Fig. 1E). Five practice trials were followed by three blocks of 48 trials. Each trial consisted of a left or right pointing arrow presented for a maximum of 1.4 s. Participants responded with a left or right finger press depending on the direction of the arrow. The arrows were centrally located and subtended a visual angle of 2.9°. The arrow disappeared as soon as participants responded. During the interstimulus interval (ISI), which was variable, there was a blank screen. There were three possible ISI durations: short (1 s ± 10% jitter); medium (2 s ± 10% jitter); and long (4 s ± 10% jitter). Each block included equal numbers of right and left trials, and equal numbers of ISI duration. The three blocks immediately followed each other so that the participant experienced a single block of 144 trials lasting approximately 9 min. RT and accuracy were calculated for each ISI duration type. As in previous work, performance in the first and last block of the task was analysed separately to test for fluctuations in vigilance level. Vigilance decrement was calculated by comparing the performance between the first and last task blocks, as in previous studies (Alexander *et al*., 2005; Malhotra *et al*., 2009; Bonnelle *et al*., 2011).

### RVP

The RVP from the Cantab battery (Cambridge Cognition, UK) probes sustained attention (Jones *et al*., 1992; Fig. 1F). Single digits (range 2–9) were sequentially presented in the centre of the screen, at a rate of 100 digits/min. Participants were required to press a button in response to the presentation of a pre‐defined sequence of numbers (3–5–7, 2–4–8, 4–6–8). Each number stimulus subtended a visual angle of 1.3°. The task lasted for 7 min, and was preceded by a 5‐min practice block. The RVP from the Cantab battery probes sustained attention (Jones *et al*., 1992; Fig. 1E). Single digits (range 2–9) were sequentially presented in the centre of the screen, at a rate of 100 digits/min. Participants were required to press a button in response to the presentation of a pre‐defined sequence of numbers (3–5–7, 2–4–8, 4–6–8). Each number stimulus subtended a visual angle of 1.3°. The task lasted for 7 min, and was preceded by a 5‐min practice block.

The following standard outcome measures were calculated (Jones *et al*., 1992; Gau & Huang, 2014; Leśniak *et al*., 2014).

1. Total misses: the number of stimuli requiring a response that are missed by the participant.
2. Probability of hits (h): total hits (correct responses) divided by the sum of total hits and total misses.
3. Total correct rejections: the number of stimuli not requiring a response that are correctly ignored by the participant.
4. Probability of false alarms (f): total false alarms (responses to inappropriate stimuli) divided by the sum of total false alarms and total correct rejections.
5. A′ (a signal detection measure of sensitivity to the target, irrespective of the participant's own tendency to respond): 0.5 + ((*h − f*)* + *(*h − f*)^2^)/(4*h*(1* − f*)).
6. B″(a signal detection measure of strength of trace required to elicit a response, that is a measure of a participant's tendency to respond to stimuli): ((*h − h*2) − (*f − f* ^2^))/((*h − h* ^2^) + (*f − f* ^2^)).
7. Latency: the RT to a correct stimulus (Sahgal, 1987; Stanislaw & Todorov, 1999; Gau & Huang, 2014).

Outcome measures were calculated for each stimulation condition. One participant missed 15 stimuli in one condition, which was > 2 SD away from the group mean for that condition. These results were excluded from further analyses.

## Results

### Stimulation modulates performance on the number comparison task

**Figure 2:**
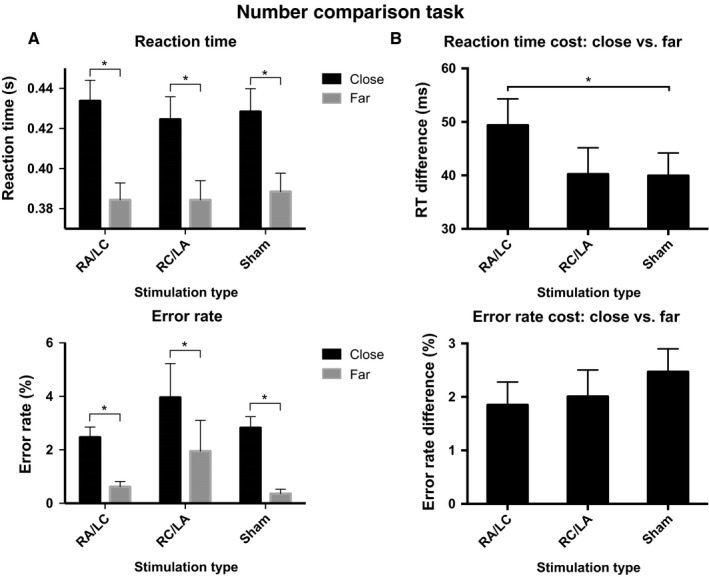
Number comparison task. (A) RTs and error rates for each stimulation condition, grouped according to trial type. (B) RT and error costs for responding to a ‘close’ number pair compared with a ‘far’ number pair, for each stimulation condition. *Denotes statistically significant Student's *t*‐test comparison (*P* < 0.05). Right anodal/left cathodal (RA/LC). Error bars denote standard error of the mean.

‘Close’ number pairs were more difficult to process than ‘far’ number pairs. This manifested as significantly slower RTs (*F*_1,17_ = 158.2, *P* < 0.001) and higher error rates (*F*_1,17_ = 48.5, *P* < 0.001) when participants responded to ‘close’ number pairs, compared with ‘far’ pairs (Fig. 2A). This processing cost was observed in all stimulation conditions.

Stimulation specifically impaired the numerical processing of more difficult stimuli, that is ‘close’ number pairs (Fig. 2B). An anova investigating the interaction of stimulation type (three levels) and number pair type (two levels) on RT showed a significant interaction (*F*_2,16_ = 3.684, *P* = 0.048). This effect was driven by an increased RT when responding to ‘close’ pairs vs. responding to ‘far’ number pairs, which was significantly greater under RA/LC stimulation compared with sham stimulation [*t* = 2.607, df = 17, *P* = 0.018, Cohen's effect size = 0.61, mean of difference = 9.43 ms (95% CI 1.78–17.06); Fig. 2B]. That is, RA/LC stimulation exaggerated the numerical processing cost of responding to ‘close’ number pairs. There was no effect of stimulation type on error rate [*F*_2,16_ = 0.747, *P* = 0.490; partial eta squared = 0.085; mean error rate for RA/LC stimulation = 3.09% (95% CI 2.18–3.99), mean error rate for RC/LA stimulation = 5.92% (95% CI 0.91–10.9), mean error rate for sham stimulation = 3.19% (95% CI 2.21–4.17)].

### Stimulation had no effect on spatial attention as assessed by the modified Posner task

**Figure 3:**
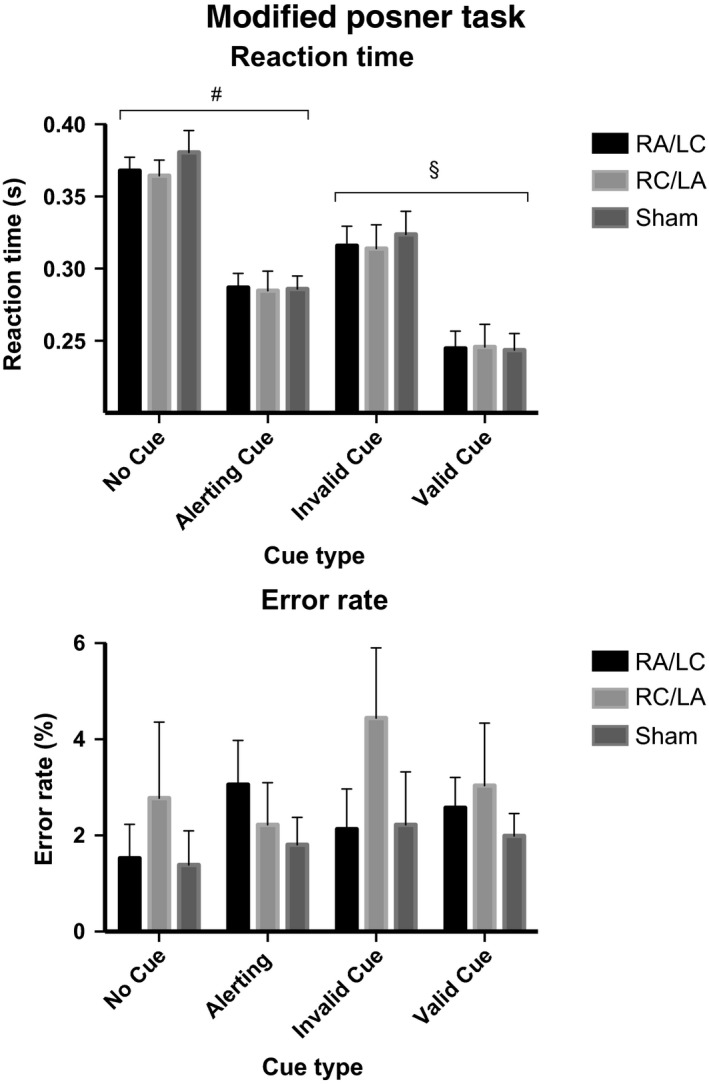
Modified Posner task: RTs and error rates are grouped according to type of cue. ^§^Indicates the Posner effect, that is a faster RT to ‘Valid cue’ trials as compared with ‘Invalid cue’ trials. ^#^Indicates the alerting effect, that is a faster RT to ‘Alerting cue’ trials as compared with ‘No cue’ trials. Error bars denote the standard error of the mean.

The modified Posner task produced the expected pattern of performance. The behavioural results were as expected. There was a main effect of cue type on RT (*F*_3,15_ = 160.35, *P* < 0.001, partial eta squared = 0.970; Fig. 3). The fastest RTs were in the valid cue condition. The RTs to ‘Valid cue’ trials were significantly faster than RTs to ‘Invalid cue’ trials (i.e. the Posner effect; *F*_1,17_ = 194.9, *P* < 0.001), ‘Alerting cue’ trials (*F*_1,17_ = 104.7, *P* < 0.001) and ‘No cue’ trials (*F*_1,17_ = 408.1, *P* < 0.001). The next fastest RTs were in ‘Alerting cue’ conditions. The RTs to ‘Alerting cue’ trials were significantly faster than RTs to ‘Invalid cue’ trials (*F*_1,17_ = 37.3, *P* < 0.001) and to ‘No cue’ trials, which is the Alerting effect (*F*_1,17_ = 37.3, *P* < 0.001). The RT to ‘Invalid cue’ trials was significantly faster than to ‘No cue’ trials, which produced the slowest RT (*F*_1,17_ = 108.1, *P* < 0.001). There was no effect of stimulation type on RT [*F*_2,16_ = 0.266, *P* = 0.770; partial eta squared = 0.032; mean RT with RA/LC stimulation = 293 ms (95% CI 271–314), mean RT with RC/LA stimulation = 291 ms (95% CI 263–320), mean RT with sham stimulation = 296 ms (95% CI 272–319)], error rate [*F*_2,16_ = 1.205, *P* = 0.326; partial eta squared = 0.131; mean error rate with RA/LA stimulation = 2.4% (95% CI 1.0–3.8), mean error rate with RC/LC stimulation = 2.9% (95% CI 0.4–5.4), mean error rate with sham stimulation = 1.8% (95% CI 0.9–2.8)] or the Posner effect [*F*_2,16_ = 1.292, *P* = 0.302; partial eta squared = 0.139; mean Posner effect with RA/LC stimulation = 71 ms (95% CI 55–87), mean Posner effect with RC/LA stimulation = 68 ms (95% CI 57–79), mean Posner effect with sham stimulation = 80 ms (95% CI 63–97)] and Alerting effects [*F*_2,16_ = 0.503, *P *= 0.614; partial eta squared = 0.059; mean alerting effect for RA/LC stimulation = 81 ms (95% CI 69–93), mean alerting effect with RC/LA stimulation = 80 ms (95% CI 65–94), mean alerting effect with sham stimulation = 95 ms (95% CI 74–116)].

Previous studies have found that oppositional parietal stimulation can promote hemispheric perceptual bias (as assessed by line bisection), although taking this into consideration in the current analysis did not reveal an effect of stimulation. A three‐way repeated‐measure anova was performed, which included the hemi‐field in which the stimulus was presented as a factor (stimulation three levels; cue four levels; hemi‐field two levels). There was an interaction between hemi‐field and cue (*F*_3,15_ = 4.126, *P* = 0.026, partial eta square = 0.452). In the alerting, valid or invalid cue conditions, responses were faster to left hemi‐field targets than right hemi‐field targets. However, in the no cue condition, responses were faster to right hemi‐field targets than to left hemi‐field targets. There was no interaction between hemi‐field and stimulation type (*F*_2,16_ = 0.625, *P* = 0.548; partial eta squared = 0.072) or between hemi‐field, stimulation and cue (*F*_6,12_ = 0.287, *P* = 0.932; partial eta squared = 0.126).

### Stimulation modulates vigilance level on the CRT

**Figure 4:**
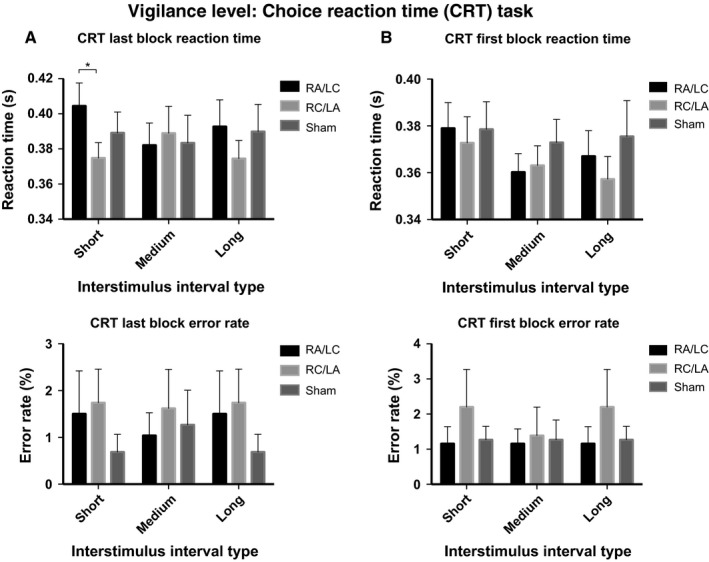
Vigilance level on the CRT: 482 RTs and error rates for the last block (A) and first block (B) of the CRT task. *Denotes statistically significant Student's *t*‐test comparison (*P* < 0.05). Error bars denote the standard error of the mean.

Stimulation had a significant effect on vigilance level, measured by RT, in the final block of the task (Fig. 4). An anova investigating the interaction of stimulation type (three levels) and ISI interval type (three levels) on RT showed a significant interaction in the last block of the task (*F*_4,14_ = 4.3, *P* = 0.018; partial eta squared = 0.551). This result was driven by the slower RT on short ISI trials, with RA/LC stimulation, as compared with RC/LA stimulation [*t* = 3.509, df = 17, *P* = 0.003; Cohen's effect size = 0.83; mean of difference = 29.7 ms (95% CI 11.9–47.6); Fig. 4A)]. This effect was not seen in the first block of the task, where there was no interaction (*F*_4,14_ = 1.626, *P* = 0.223; partial eta squared = 0.317) and no main effects of stimulation type [*F*_2,16_ = 2.905, *P* = 0.084; mean RT with RA/LC stimulation = 369 ms (95% CI 349–388), mean RT with RC/LA stimulation = 364 ms (95% CI 345–389), mean RT with sham stimulation = 376 ms (95% CI 351–401)] or ISI type (*F*_2,16_ = 3.119, *P* = 0.072) on RT. There was no effect of stimulation on accuracy in either the first block [*F*_2,16_ = 1.049, *P* = 0.373; mean error rate with RA/LC stimulation = 2.4% (95% CI 1.2–3.7), mean error rate with RC/LA stimulation = 2.3% (95% CI 1.5–3.2), mean error rate with sham stimulation = 2.1% (95% CI 1.5–2.7)] or the last block of the CRT [*F*_2,16_ = 0.082, *P* = 0.922; mean error rate with RA/LC stimulation = 0.5% (95% CI −0.2 to 1.1), mean error rate with RC/LA = 0.6% (95% CI 0.0–1.2), mean error rate with sham stimulation = 0.7% (95% CI −0.1 to 1.6)].

**Figure 5:**
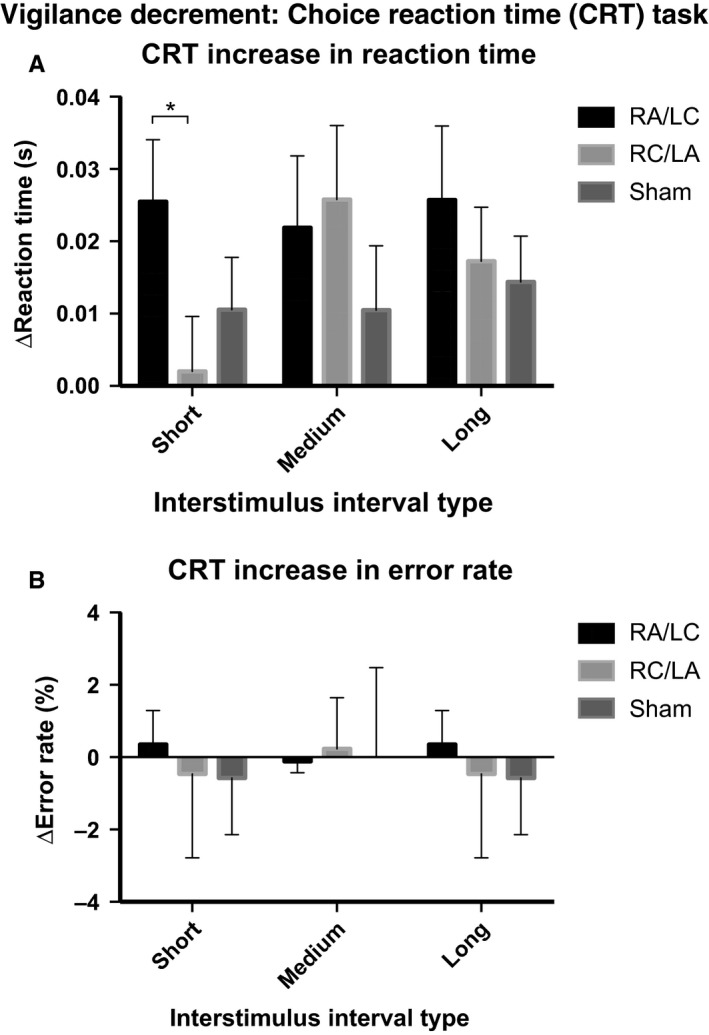
Vigilance decrement on the CRT: RT change (A) and error rate change (B). *Denotes statistically significant Student's *t*‐test comparison (*P* < 0.05). Error bars denote the standard error of the mean.

Vigilance decrement was calculated as an increased RT between the first and last blocks of the CRT (as in Bonnelle *et al*., 2011). anova was used to investigate stimulation type and ISI interval on this measure. This showed an interaction of borderline significance (*F*_4,14_ = 2.66, *P* = 0.077; partial eta squared = 0.432), driven by a borderline effect of stimulation on RT increase in short ISI trials (*F*_2,16_ = 3.249, *P* = 0.065; partial eta squared = 0.289). Specifically, the RT increase with RA/LC stimulation was significantly greater than the RT increase with RC/LA stimulation [*t* = 2.28, df = 17, *P* = 0.036; Cohen's effect size = 0.54; mean of difference = 23.5 ms (95% CI 17.5–45.1); Fig. 5A]. This is consistent with either decreased vigilance level in the final block of the task with RA/LC stimulation or an improvement in vigilance level with RC/LA stimulation. Overall, there was no main effect of trial type (*F*_2,16_ = 0.764, *P* = 0.482, partial eta squared = 0.087) and no effect of trial type when the three stimulation conditions were tested separately (*P* > 0.1), despite the short ISI condition having a negligible vigilance decrement in the RC/LA condition. For errors, there was no interaction (*F*_2,16_ = 0.497, *P* = 0.618; partial eta squared = 0.058), main effect of stimulation type [*F*_2,16_ = 0.618, *P* = 0.551; partial eta squared = 0.072; mean change in error rate with RA/LC stimulation = 0.2% (95% CI −1.3 to 1.6), mean change in error rate with RC/LA stimulation = −0.2% (95% CI −1.0 to 0.6), mean change in error rate with sham stimulation = −0.4% (95% CI −1.1 to 0.4)] or effect of ISI duration (*F*_1,17_ = 0.452, *P* = 0.510; partial eta squared = 0.026).

There was no correlation between the effect of stimulation on vigilance level and the effect of stimulation on the numerical processing cost of responding to close vs. far number pairs (Spearman's correlation: *r*_s_ = 0.282, *P* = 0.257).

### RVP

**Figure 6:**
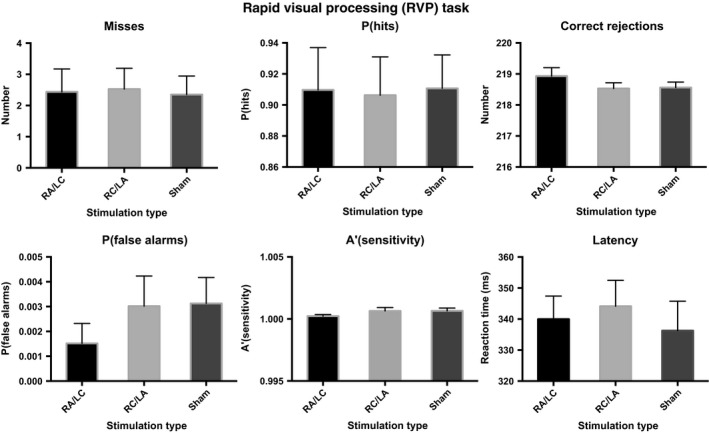
RVP outcome measures, detailed in the main text, as a function of stimulation type. Error bars denote the standard error of the mean.

There was no significant effect of stimulation on any of the outcome measures: number of misses [*F*_2,16_ = 0.235, *P* = 0.793; partial eta squared = 0.029; mean with RA/LC stimulation = 3.0 (95% CI 0.9–5.0), mean with RC/LA stimulation = 2.9 (95% CI 1.4–4.4), mean with sham stimulation = 2.5 (95% CI 1.3–3.7)]; probability of correct response [*P*(hits)] [*F*_2,15_ = 0.169, *P* = 0.846, partial eta squared = 0.022; mean with RA/LC stimulation = 0.88 (95% CI 0.80–0.96), mean with RC/LA stimulation = 0.90 (95% CI 0.84–0.96), mean with sham stimulation = 0.90 (95% CI 0.86–0.95)]; number of correct rejections [*F*_2,16_ = 0.60, *P* = 0.561; partial eta squared = 0.07; mean with RA/LC stimulation = 218.8 (95% CI 218.1–219.3), mean with RC/LA stimulation = 218.5 (95% CI 218.1–218.9), mean with sham stimulation = 218.6 (95% CI 218.3–219.0)]; probability of false alarms [*P*(false alarms)] [*F*_2,16_ = 0.087, *P* = 0.917; partial eta squared = 0.011; mean with RA/LC stimulation = 0.3 (95% CI 0.0–0.6), mean with RC/LA stimulation = 0.3 (95% CI 0.1–0.6), mean with sham stimulation = 0.3 (95% CI 0.1–0.5)]; sensitivity to stimuli (A′) [*F*_2,16_ = 1.405, *P* = 0.274; partial eta squared = 0.149; mean with RA/LC stimulation = 1.000 (95% CI 1.000–1.001), mean with RC/LA stimulation = 1.001 (95% CI 1.000–1.001), mean with sham stimulation = 1.001 (95% CI 1.000–1.001)]; and RT [*F*_2,15_ = 0.769, *P* = 0.481; partial eta squared = 0.093; mean with RA/LC stimulation = 343.5 ms (95% CI 326.5–360.5), mean with RC/LA stimulation = 349.6 ms (95% CI 327.1–372.1), mean with sham stimulation = 342.7 ms (95% CI 319.6–365.8); Fig. 6]. It was not possible to calculate group values for strength of trace (B′) because, for some participants, the denominator was 0.

## Discussion

It was shown that oppositional tDCS applied across the parietal lobes can affect numerial processing and sustained attention in a way that depends on the polarity of the stimulation. RA/LC stimulation exaggerated the distance effect in performance of a number comparison task, compared with sham stimulation. In addition, parietal stimulation had a polarity‐dependent effect on vigilance level, when stimulation conditions were directly compared. Furthermore, the effects of tDCS were only observed when cognitive load was high, that is during more demanding numerical processing and when attentional demands were increased because of prolonged task performance.

The P3/P4 electrode positions used here overlie the inferior parietal cortex (Kim *et al*., 2007). Therefore, the most straightforward explanation of why RA/LC tDCS impaired performance on the number comparison task is that cathodal stimulation inhibited the left inferior parietal lobule/angular gyrus, which subserve numerical processing (Göbel *et al*., 2001; Sandrini *et al*., 2012). Lesions within the left hemisphere often produce impairments of numerical processing (Zukic *et al*., 2012), and transcranial magnetic stimulation (TMS) studies have shown that inhibitory TMS applied to the left parietal lobe can disrupt performance on number comparison tasks (Göbel *et al*., 2001; Sandrini *et al*., 2012; Andres *et al*., 2005; Cappelletti *et al*., 2008). Additionally, left anodal parietal stimulation has been shown to improve accuracy on a number comparison task (Hauser *et al*., 2013).

However, this interpretation may be too simplistic because numerical comparison tasks involving numbers, number words and even non‐symbolic representations of numbers, such as collections of dots, consistently activate ‘bilateral’ posterior parietal cortices (Pinel *et al*., 2001; Ansari *et al*., 2006; Notebaert *et al*., 2010). Furthermore, a behavioural and physiological distance effect is also observed in such numerical comparison tasks, with smaller numerical distances between stimuli resulting in stronger biparietal activation (Pinel *et al*., 2001; Ansari *et al*., 2006; Notebaert *et al*., 2010), as well as slower and less accurate responses (Moyer & Landauer, 1967; Dehaene *et al*., 1990). The task‐related activation also reflects the ratio of numerical distance and absolute magnitude of the numbers (Piazza *et al*., 2004). This suggests that bilateral regions subserve processing of numerical distance. This led Hauser and colleagues to apply parietal bi‐anodal (i.e. not oppositional) tDCS during a number comparison task. Although left anodal tDCS improved overall accuracy, neither the bi‐parietal nor the unilateral montages modulated the distance effect (Hauser *et al*., 2013). In contrast, it was possible to selectively modulate the distance effect using an oppositional RC/LA montage. This suggests that the left and right posterior parietal cortex support distinct aspects of numerical processing, as suggested by others (Dehaene *et al*., 1996; Chochon *et al*., 1999; Mussolin *et al*., 2013). It has been postulated that the left inferior parietal sulcus has greater precision in numerical coding (Piazza *et al*., 2004; Andres *et al*., 2005), possibly because of its left‐sided language networks underlying verbal coding of numbers (Dehaene, 2001). Furthermore, there may be an element of interhemispheric inhibition between parietal hemispheres, as a study of inhibitory rTMS of the intraparietal sulcus found impaired number comparison performance with left rTMS but improved performance after right‐sided rTMS (Cappelletti *et al*., 2008). An asymmetrical and adversarial component to numerical processing by the parietal lobes might explain why the particular montage (oppositional RA/LC) produced effects on the distance effect on the number comparison task.

Also, a distinct polarity‐dependent effect of parietal tDCS on both vigilance level and decrement was shown. Comparing the stimulation montages directly showed that RTs for a simple CRT were greater for the RA/LC than RC/LA montage at the end of the task. This shows that distinct types of oppositional parietal tDCS can modulate vigilance. However, the effects of both montages were not significantly different from the sham condition. Therefore, it is not possible to be certain whether the RA/LC montage impaired vigilance and/or the RC/LA montage enhanced it. Further work will be necessary to answer this question.

A bi‐parietal oppositional montage thus appears to affect both vigilance and numerical processing. Therefore, the observed effects of tDCS on the number comparison task could be partially explained by the effects of tDCS on sustained attention or a combination of specific effects on numerical processing and non‐specific attentional effects. In keeping with a common effect, Husain and colleagues have previously argued that spatial neglect following right parietal stroke results from a combination of a specific impairment of spatial processing and a non‐specific effect on sustained attention (Husain *et al*., 1997; Husain & Rorden, 2003). This possibility is also supported by a recent tDCS study that showed improved working memory performance by using a parietal montage that aimed to simultaneously boost selective attention (anodal tDCS of the left intraparietal sulcus/superior parietal lobule) and diminish spatial attention (using cathodal tDCS of the right inferior parietal cortex; Jacobson *et al*., 2012b). This demonstrated that working memory performance could be modulated by manipulating different aspects of attention (Jacobson *et al*., 2012b). In the current study, there was no correlation in change in performance on the number comparison and CRT with RA/LC stimulation, suggesting that the effect on attention is only one of a number of other possible contributing factors. Future studies are required to probe this possible interaction between attention and numerical processing.

The effect of tDCS was only detectable when task demands were high, either because of the need for sustained task performance (CRT) or because decisions were made on numbers that were ‘close’ rather than ‘far’. This suggests an interaction between the effects of parietal tDCS and cognitive load. In addition, a distinct effect of tDCS on vigilance was only seen when the event rate was high, that is when the gap between stimuli was short. This could also reflect an interaction between task demands and stimulation, as some studies have suggested that fast event rates demand more vigilance than slower event rates (Parasuraman, 1979; Sarter *et al*., 2001). This interaction between task demand and stimulation may also help to explain why RA/LC stimulation resulted in lower vigilance than RC/LA stimulation, a finding that seems to contradict the accepted understanding of sustained attention being subserved by right parietal regions (for review, see Singh‐Curry & Husain, 2009). A previous TMS study found that inhibition of either the left or right inferior parietal lobe impaired vigilance decrement in a spatial sustained attention task, suggesting that the left hemisphere is also involved in maintaining attention (Lee *et al*., 2013). Additional evidence for this possibility is provided by Helton and colleagues who used near‐infrared spectroscopy to study cerebral blood flow during an easy and a more difficult sustained attention task. They found that whilst right parietal hemisphere blood flow was predominant in the easy task, when participants performed the harder task, blood flow increased in both the left and right parietal hemispheres (Helton *et al*., 2010).

This type of interaction between brain stimulation and cognitive load has only previously been reported in the context of working memory, to the authors’ knowledge (Jones & Berryhill, 2012; Sandrini *et al*., 2004; Wu *et al*., 2014). For example, using an oppositional montage, Sandrini *et al*. (2004) showed the electrode polarity that RA/LC stimulation abolished the effect of familiarity on working memory performance when the task was difficult. In contrast, the reverse effect (i.e. disruption of familiarity effect when the task was easy) was produced with opposite polarity stimulation (RC/LA). One possible interpretation of these findings is that hemispheric interactions, modulated by oppositional stimulation, may become particularly important under high task demands where the effects of activity in the other hemisphere may be more disruptive.

An influence of cognitive load might partly explain why results of brain stimulation on cognitive tasks are so variable. A recent meta‐analysis found that the probability of finding the anodal‐facilitatory/cathodal‐inhibitory effect on behaviour in cognitive studies was low. In particular, cathodal‐inhibitory effects were difficult to produce (Jacobson *et al*., 2012a). One reason for this is that inhibition of a single region by cathodal tDCS might usually be insufficient to impair functions that are supported by distributed cortical networks. However, by applying cathodal tDCS during a cognitively demanding task, the effects on the whole network might be enough to impair behaviour due to a baseline reduction of available cognitive resources. The current findings suggest that future studies should consider in more detail the relationship between task features, attentional demands and stimulation. Oppositional montages may be well suited to investigate these effects, especially if hemispheric interactions are thought to be important (Cohen Kadosh *et al*., 2010; Hecht *et al*., 2010; Sandrini *et al*., 2004; Bardi *et al*., 2013). An extension of this study would be to investigate how baseline performance influences the response to stimulation, which has been seen in spatial attention amongst other motor and cognitive functions (Benwell *et al*., 2015; Li *et al*., 2015), as one's baseline performance may be related to how one handles cognitive load.

This study did not show any effect on spatial attention measured using the Posner task. Within the parietal lobe, spatial attention is thought to involve interactions between the right superior parietal lobule and bilateral intraparietal sulci (Corbetta & Shulman, 2002, 2011). The involvement of the right superior parietal lobule is why lesions of the right parietal lobe after stroke result in neglect. Interhemispheric competition between the bilateral intraparietal sulcus is also thought to underlie efficient coding of stimulus location, with each parietal hemisphere favouring processing of stimuli in the contralateral hemi‐field (Kinsbourne, 1987; Sylvester *et al*., 2007). Clear Posner and Alerting effects were shown, indicating that participants were performing the task appropriately (Posner *et al*., 1980; Fan *et al*., 2002). Therefore, the current finding that oppositional parietal tDCS had no effect on spatial attention was unexpected, particularly as previous studies have demonstrated that RC/LA parietal (P3/P4) stimulation produces a rightward bias in a centroid defining task (Wright & Krekelberg, 2014) and non‐manual line bisection tasks (Giglia *et al*., 2011; Benwell *et al*., 2015). This lack of effect of stimulation may be due to a lack of power. However, it may also be due to key differences in the attentional systems required when performing the Posner task compared with the line bisection or centroid finding tasks. A previous study that investigated clinical measures of neglect, including manual line bisection, did not find that performance in this correlated to performance in the Posner task (Siéroff *et al*., 2007). A key component in the Posner task is the ‘re‐orientation’ of attention, which may require bilateral parietal involvement (Vossel *et al*., 2009; Doricchi *et al*., 2010). It is therefore possible that an oppositional montage in this task produced competing cortical effects within bilateral parietal areas, which reduced any overall effect, although future experiments would be needed to clarify this.

The current study has a number of limitations. The challenges of interpreting the physiological basis of the effects of oppositional montages were discussed, and future studies could dissect these by including control conditions that include stimulation directed to only one hemisphere. In addition, although this study was adequately powered to detect the effect of stimulation on some cognitive tasks, it may have been underpowered to detect the effect on all tasks because of different levels of test–retest variability across the tasks used. The order of tasks was the same throughout the study, so a potential confound is the timing of tasks relative to stimulation onset. The potential for task timing to confound results presents an important question for future studies to address. To the authors’ knowledge this has not been investigated extensively in the literature, even in the motor system (Stagg *et al*., 2011), and so it is unclear how problematic this factor is. Another potential confound is task duration. It could be that a task did not show behavioural effect because it was not performed for sufficient duration for tDCS to modulate relevant networks. An effect of stimulation on the shortest task (number comparison task) was found; however, it could be that the duration required for tDCS to be effective is task dependent, which is a question that merits further investiation. Finally, although cognitive performance could be impaired, this study failed to improve performance with tDCS of the opposite polarity. For some of the tasks this may have been due to a ceiling effect of performance, and future studies might have more power to detect improvements by either using more difficult tasks or studying patient populations who show baseline cognitive impairments (Bonnelle *et al*., 2011; Kang *et al*., 2012; Nelson *et al*., 2013; Mcintire *et al*., 2014).

In summary, this study demonstrated that oppositional parietal stimulation modulates numerical processing and vigilance level in a polarity‐dependent manner. The effect was only observed in situations of relatively high cognitive load, suggesting that the impact of oppositional tDCS on hemispheric interactions depends on task difficulty as well as specific processing demands and attentional effects.
